# Use of point-of-care ultrasound (POCUS) by emergency physicians for general surgical patients in resuscitation room

**DOI:** 10.1186/2036-7902-6-S1-A20

**Published:** 2014-01-31

**Authors:** KH Cheung, YS Ong, CA Graham, TH Rainer, NK Cheung

**Affiliations:** 1 Emergency Department, Prince of Wales Hospital, Shatin, Hong Kong; 2 Accident and Emergency Medicine Academic Unit, Chinese University of Hong Kong

## Background

Point-of-care ultrasound (POCUS) by emergency physicians (EP) is very useful for giving timely diagnosis. Applications in surgical patients include FAST/ EFAST in trauma settings, abdominal ultrasound (e.g. abdominal aortic aneurysm, hepatobiliary ultrasound), shock assessment (RUSH protocol), and ultrasound-guided vascular access.

## Objective

To assess the utilization rate and diagnostic performance of POCUS by EP for critically ill surgical patients in a tertiary hospital.

## Patients and methods

Patients having general surgical presentation (i.e. excluding patients with neurosurgical, urological or cardiothoracic chief complaints) who attended Emergency Department of Prince of Wales Hospital, and triaged as category I or II (emergency or urgent) were included. The inclusion period was from Sep 2012 to Mar 2013. Patients’ clinical records were retrieved. Documented POCUS and scope of use by EP, yield to diagnosis, and eventual diagnoses after admission were studied.

## Results

A total of 98 patients were included within the half-year period. POCUS by EP were performed in 34 patients (34.7%). Among all patients 19 (19.4%) were trauma cases while 79 (80.6%) were non-traumatic. For trauma patients, FAST/ EFAST were performed in 17 of them (89.4%). Among non-trauma patients, focused abdominal ultrasound was performed in 16 patients (20.3%), out of which 4 patients (5%) had abdominal aortic aneurysm detected. Intra-peritoneal free fluid was detected in 3 patients (3.8% of non-traumatic patients), which later turned out to be ruptured hepatocellular carcinoma. 2 patients had stone in common bile ducts detected, confirming biliary sepsis; and 1 patient had liver cyst detected. When compared with CT results or discharge diagnosis, the sensitivity, specificity, positive predictive value and negative predictive value of EP POCUS was 86%, 94%, 92% and 88% respectively.

## Conclusion

Utilization rate of POCUS by EP on critically ill surgical patients in ED was 34.7%. Diagnostic performance of EP POCUS was reasonably good.

